# A compact system for intraoperative specimen imaging based on edge illumination x-ray phase contrast

**DOI:** 10.1088/1361-6560/ab4912

**Published:** 2019-11-26

**Authors:** Glafkos Havariyoun, Fabio A Vittoria, Charlotte K Hagen, Dario Basta, Gibril K Kallon, Marco Endrizzi, Lorenzo Massimi, Peter Munro, Sam Hawker, Bennie Smit, Alberto Astolfo, Oliver J Larkin, Richard M Waltham, Zoheb Shah, Stephen W Duffy, Rachel L Nelan, Anthony Peel, Tamara Suaris, J Louise Jones, Ian G Haig, David Bate, Alessandro Olivo

**Affiliations:** 1 Department of Medical Physics and Bioengineering, University College London, WC1E 6BT, United Kingdom; 2 Nikon X-Tek Systems, Tring Business Centre, Icknield Way, Tring, Hertfordshire, HP23 4JX, United Kingdom; 3 Barts and the London School of Medicine and Dentistry, Queen Mary University of London, Newark St, London E1 2AT, United Kingdom; 4 St Bartholomew’s Hospital, Barts Health NHS Trust, West Smithfields, London EC1A 7BE, United Kingdom; 5 Current address: ENEA- Radiation Protection Institue, 4 Via Martiri di Monte Sole, 40129 Bologna, Italy; 6 Author to whom correspondence should be addressed.; glafcos.havariyoun.10@ucl.ac.uk

**Keywords:** phase contrast imaging, intraoperative imaging, x-ray phase contrast imaging, CT phase contrast imaging, edge illumination phase contrast imaging, x-ray imaging

## Abstract

A significant number of patients receiving breast-conserving surgery (BCS) for invasive carcinoma and ductal carcinoma *in situ* (DCIS) may need reoperation following tumor-positive margins from final histopathology tests. All current intraoperative margin assessment modalities have specific limitations. As a first step towards the development of a compact system for intraoperative specimen imaging based on edge illumination x-ray phase contrast, we prove that the system’s dimensions can be reduced without affecting imaging performance.

We analysed the variation in noise and contrast to noise ratio (CNR) with decreasing system length using the edge illumination x-ray phase contrast imaging setup. Two-(planar) and three-(computed tomography (CT)) dimensional imaging acquisitions of custom phantoms and a breast tissue specimen were made. Dedicated phase retrieval algorithms were used to separate refraction and absorption signals. A ‘single-shot’ retrieval method was also used, to retrieve thickness map images, due to its simple acquisition procedure and reduced acquisition times. Experimental results were compared to numerical simulations where appropriate.

The relative contribution of dark noise signal in integrating detectors is significant for low photon count statistics acquisitions. Under constant exposure factors and magnification, a more compact system provides an increase in CNR. Superior CNR results were obtained for refraction and thickness map images when compared to absorption images. Results indicate that the ‘single-shot’ acquisition method is preferable for a compact CT intraoperative specimen scanner; it allows for shorter acquisition times and its combination of the absorption and refraction signals ultimately leads to a higher contrast. The first CT images of a breast specimen acquired with the compact system provided promising results when compared to those of the longer length system.

## 1. Introduction

### 1.1. Background

One in eight women are affected by breast cancer in their lifetime (Smittenaar *et al* 2016). For early-stage breast cancer, breast-conserving surgery (BCS) and therapy is the preferred standard of care (NICE 2018). The success of BCS is dependent on the excision of the tumour with an adequate but limited margin of healthy tissue. Re-excision is required when histopathology-based post-operative tests indicate that either the tumour is too close to the specimen margins or the tumour infiltrates the margins. Re-excision rates vary from centre to centre; a recent prospective study indicated a 0% to 41% (median 17.2%) re-excision rate across 76 individual centres in the UK (Tang *et al* 2017). Re-operation can cause patients additional stress and affect cosmetic outcome. Furthermore, the additional time and cost implications are significant to the healthcare system.

### 1.2. Tumour margin assessment techniques

For impalpable masses, most centres in the UK use simple planar x-ray radiography systems to assess intra-operatively if the margins of the resected lump are clear (John *et al* 2017). However, this is not adequate for conditions such as ductal carcinoma *in situ* (DCIS), invasive lobular carcinoma and microscopic margin involvement, due to shortcomings of conventional x-ray imaging which are mainly due to its dependence on absorption effects generated by differences in attenuation coefficients of tissues of interest.

Other margin involvement assessment methods, during primary surgery, include intraoperative ultrasonography (IOUS) and pathology assessment with frozen section (FS) or touch imprint cytology (TIC) (John *et al* 2017). IOUS shares the same limitations as planar x-ray radiography (i.e. similarities in tissue densities) and also requires breast surgeons to train and qualify in intraoperative US-guided surgery. FS and TIC methods have the highest sensitivity and specificity rates but they are time consuming, labour intensive and require availability of pathology staff. FS, requiring approximately 30 min, involves freezing and sectioning of the specimen followed by thawing, fixation and staining. The freezing and thawing of specimens may lead to artefacts and tissue loss (Laucirica 2005). TIC, a simpler method which requires approximately 15 min, involves pressing all margins of the excised specimen onto glass sides which are then fixed and stained (Singletary 2002). This is a local assessment method (i.e only assesses the surface of the specimen) which is linked to errors due to surface irregularities, specimen size, presence of atypical cells, dryness and the pathologist’s interpretation skills (Weinberg *et al* 2004, Laucirica 2005).

### 1.3. Emerging tumour margin assessment techniques

In light of the limitations of the above methods and the need to reduce re-excision rates, several new technologies have been developed over recent years. In the imaging category, the feasibility of intraoperative micro-computed tomography has been investigated in a few studies with limited number of samples and scan times of up to 7 min. However, despite resolution values of 4–50 *µ*m, this x-ray imaging technique is also hampered by the very similar densities of cancer and healthy breast parenchyma in patients with dense breasts. Although a small number of optical imaging studies such as Raman spectroscopy (Kong *et al* 2014) and optical coherence tomography (Nguyen *et al* 2009) have reported high sensitivity and specificity values, these assessment methods suffer from long image acquisition times, limited depth penetration, artefacts from cauterised tissue and penetration of dyes into healthy tissue. Moreover these methods are not volumetric and only allow to probe the specimen at specific locations making the probing of the entire margin difficult. Radiofrequency methods exploit tissue-specific spectral signatures by exposing tissue to an electric field (Karni *et al* 2007, Dixon *et al* 2015). Although these methods are sensitive to cellular and molecular features of cancer on the surface of resected tissue, they are unable to produce high-resolution volumetric images. One of the most recent techniques, based on mass spectrometry, involves the analysis of the electrosurgical plume of diathermy smoke to determine the structural lipid profile of tissue. A preliminary proof of concept study has indicated that not only does this rapid evaporative ionisation mass spectroscopy (REIM) method have high sensitivity and specificity rates, but it also does not disrupt workflow in the theatre (St John *et al* 2017). However, due to the unique signature of each cell type, further validation is required to assess its diagnostic accuracy with more rare cancer types.

### 1.4. X-ray phase contrast imaging: edge illumination

X-ray phase contrast imaging (XPCI) extends the capabilities of absorption based imaging due to its sensitivity to phase-shifts introduced by the sample as the beam traverses it. XPCI has been shown to provide superior image contrast when compared to conventional absorption based x-ray imaging, especially for materials composed of low atomic number elements, such as soft biological tissues (Bravin *et al* 2012). A sample’s absorption and refraction properties are described by its complex refractive index:

1 \begin{align*} \newcommand{\e}{{\rm e}} \displaystyle n\left(E \right)=1-\delta \left(E \right)+i\beta \left(E \right)~\nonumber \end{align*}

where *E* is the photon energy, the imaginary part *β* is related to the absorption properties and the unit decrement of the real part *δ* refers to the phase shift of the x-ray beam. Consequently the terms:

2 \begin{align*} \newcommand{\e}{{\rm e}} \displaystyle \mu \left(x,y;E \right)=2k\cdot \int_{s}{\beta \left(x,y,z;E \right)}dz~\nonumber \end{align*}

3 \begin{align*} \newcommand{\e}{{\rm e}} \displaystyle \Phi \left(x,y;E \right)=k\cdot \int_{s}{\delta \left(x,y,z;E \right)}dz~\nonumber \end{align*}

where $k$ is the wavenumber describe the absorption (*µ*) and total phase shift (Φ) introduced by a sample (*s*) on a beam with energy *E* (Paganin 2006).

Edge illumination (EI) is a non-interferometric XPCI technique that has been undergoing extensive development at University College London (Olivo *et al* 2001, Olivo and Speller 2007a). EI has been successfully implemented with both synchotron and conventional laboratory radiation sources (Munro *et al* 2012, Diemoz *et al* 2013). The method allows for quantitative retrieval of both absorption, refraction and ultra-small angle-scattering signals (i.e. dark field imaging) from a sample (Endrizzi and Olivo 2014, Endrizzi *et al* 2014). More recently, the method has been extended to quantitative computed tomography (CT) imaging (Hagen *et al* 2014a, 2014b, Zamir *et al* 2016).

EI has proven its ability to work accurately with spatially and temporally incoherent x-ray sources (Munro *et al* 2012), reduced exposure times (Olivo *et al* 2011), higher x-ray energies, relatively flexible setup requirements and a large field of view (FOV) (Endrizzi *et al* 2015, Zamir *et al* 2016, Astolfo *et al* 2017). Following further development, EI would therefore be suitable for a series of applications, among which our focus here will be on a compact system for intra-operative specimen imaging.

**Figure 1.**
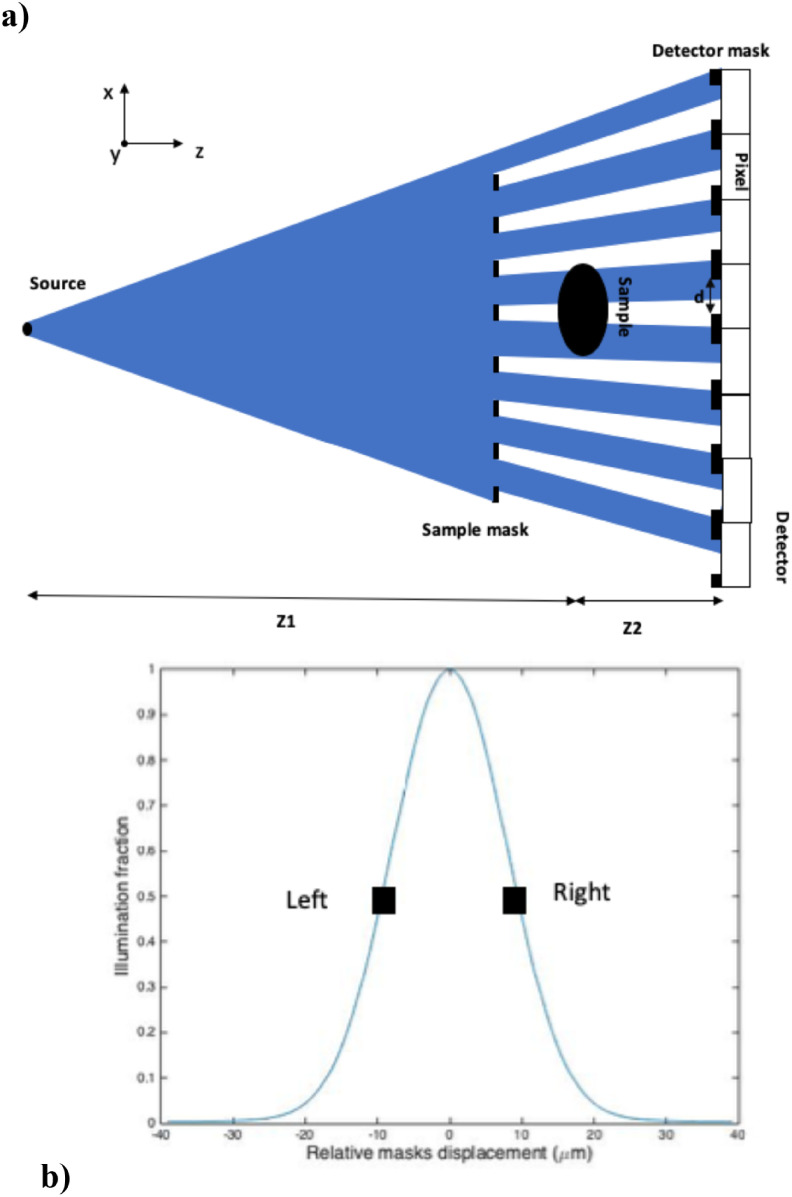
(a) Schematic diagram (not to scale) of the edge illumination experimental setup with a conventional laboratory x-ray source. (b) Example of illumination curve showing the normalised intensity variation as a function of sample mask displacement without a sample. Squares indicate the mask displacement values typically used for image acquisition.

One of the challenges for a clinical implementation is the overall system size. The standard laboratory based setup used by our group is 2 m long. Whilst not impossible, such a system length might not be ideal and convenient for clinical use. In this work we present simulation and experimental data indicating the viability of shorter EI setups for two (2D) and three-dimensional (3D), i.e. CT imaging. Figure 1 shows a schematic of the laboratory implementation of EI XPCI. For a detailed explanation of the method’s principles, the reader is referred to previous publications (Olivo and Speller 2007a, Diemoz *et al* 2013b, Hagen *et al* 2014b).

To understand how the exploitation of phase effects increases contrast, consider the ray tracing description of x-rays, where a distortion of the incoming wave front would locally be equivalent to a small deviation of individual photons from their original path, otherwise known as refraction. The angle of deviation (refraction angle), is proportional to the local variation in the phase shift, and is given by

4 \begin{align*} \newcommand{\e}{{\rm e}} \displaystyle \alpha \left(x,y;E \right)\cong {{k}^{-1}}\left| \nabla \Phi \left(x,y;E \right) \right|.\nonumber \end{align*}

Hence, as the angle of refraction is proportional to the gradient of the phase shift, Φ, it is largest at the boundaries of objects or details within it (Endrizzi *et al* 2014). Angular resolution in differential phase-contrast imaging provides an estimate of the lowest detectable refraction angle, and hence an indication of the system’s sensitivity; this value can be estimated through noise measurements in the refraction angle image (Modregger *et al* 2011). Under the assumption of pure statistical (Poissonian) noise in the acquired images, the dependence of angular resolution on the number of detected photons, the propagation distance and beam profile at the detector plane has already been shown to be given by Diemoz *et al* (2013):

5 \begin{align*} \newcommand{\e}{{\rm e}} \displaystyle \sigma \left(\Delta {{\alpha }_{x,eff}} \right)\cong ~\frac{\sqrt{C({{x}_{e}})}}{{{z}_{2}}\sqrt{2{{T}_{eff}}{{I}_{0}}}[{{\rho }_{ref}}\left({{x}_{e}} \right)-{{\rho }_{ref}}\left({{x}_{e}}+d \right)]}\nonumber \end{align*}

where *σ*(Δ*α*_*x,eff*_) is the standard deviation of the effective refraction angle, *z*_2_ is the sample to detector distance, *T*_*eff*_ is the object transmission, *I*_0_ is the beam incident on the sample, *ρ*_*ref*_ is the normalised spatial distribution of the beam incident on the detector mask in the absence of the sample and *d* is the size of the aperture. *C*(*x*_*e*_) is the fraction of photons that is transmitted through the detector mask as a function of the mask displacement *x*_*e*_. This is the so-called illumination function and its values range from approximately 0 (for completely misaligned masks) to 1 (for aligned masks) (see figure 1(b)). Note that equation (5) is valid for refraction images reconstructed from two images acquired at two symmetric points on the illumination function. In an EI setup where magnification, exposure parameters and masks are kept constant, the only parameters in the above function that would vary with increasing system length are *z*_2_ and *I*_0_. As a result of the inverse square law, the number of photons incident on the detector per unit time is inversely proportional to the square of the distance between the source and detector (i.e. ${{I}_{{\rm 0}}}\propto {\rm 1}/{{\left({{z}_{{\rm 1}}}+ {{z}_{{\rm 2}}} \right)}^{{\rm 2}}}$. As *I*_0_ is under square root in equation (5), the increase in *σ*(Δ*α*_*x*,*eff*_) (i.e. worsening angular sensitivity) with a decreasing *z*_2_ would be compensated by a corresponding increase in the number of photons incident on the detector. Reducing the system length at a constant magnification inevitably leads to a smaller source to sample distance and therefore to an increase in radiation dose for a given exposure time, but this would not be an issue for the *ex vivo* imaging of tissue specimens and could be used to perform faster acquisitions.

## 2. Materials and methods

### 2.1. 2D (planar) acquisitions

2D acquisitions were made with the total system length (*z*_1_  +  *z*_2_) being increased from 1 m to 3 m in 0.5 m increments and constant magnification. The sample mask period was 79 *µ*m with an aperture size of 10 *µ*m, while the detector mask period and aperture size were 98 *µ*m and 17 *µ*m, respectively. The two masks were misaligned along the x-axis in order to acquire images at ‘50% illumination fraction’ (Olivo and Speller 2007b). Acquisitions were performed under opposing edge illumination conditions (i.e. left and right side of the illumination curve (IC) (see figure 1(b))) to enable quantitative separation of the refraction (differential) and attenuation signals (Diemoz *et al* 2013). Furthermore, in order to satisfy fast image acquisition and reconstruction requirements for a potential CT intra-operative scanner, a second, faster image reconstruction method was used to directly retrieve the sample thickness map from a single EI image (i.e. from acquisitions performed only on one slope of the IC) (Diemoz *et al* 2016). Hence, all refraction and absorption images presented in this work were reconstructed using data from opposing edge illumination conditions, and thickness map images were reconstructed using images from only one of the slopes of the IC.

The source used for the 2D acquisitions was a Rigaku MultiMax-9 rotating anode tube (Rigaku Corporation, Japan) with a tungsten anode and an effective focal spot size of approximately 70 *µ*m. All 2D acquisitions were made with a source voltage of 40 kVp and current of 10 mA. The exposure time (1.2 s per frame) was kept constant for all system configurations. The detector was a CMOS-based flat panel C9732DK-11 (Hamamatsu, Japan) with directly deposited CsI and a 50  ×  50 *µ*m^2^ pixel size. Due to the ‘line-skipping’ design of the detector mask, in which every other detector column is illuminated, the pixel size in the x-direction is effectively 100 *µ*m. Hence, data from every other pixel column was discarded during the image reconstruction process. The sample was shifted 16 times in steps of 5 *µ*m along the horizontal direction (x-axis), and 10 frames were acquired at each position to improve image statistics. This ‘dithering’ process involves combining all the frames to reconstruct an image with higher spatial resolution (Diemoz *et al* 2015). The total acquisition time was 6.4 min. This is mostly due to the extensive dithering that was implemented to sample the refraction peaks as carefully as possible; this would not be required in an intraoperative scan.

It is known that integrating detectors, like the one we used, are subject to a dark noise signal (DNS), which varies with the integration time (Endrizzi *et al* 2013). The effect of DNS on our imaging method was modelled and compared to experimental data. Simulations were performed using a wave optics simulation of the EI setup (Vittoria *et al* 2013). A constant DNS ($\sigma _{dark}^{2})$ component, due to the constant integration time used experimentally, was added in quadrature to the simulation’s noise model (photon quantum (Poisson) noise ($\sigma _{Poisson}^{2}$))

6 \begin{align*} \newcommand{\e}{{\rm e}} \displaystyle \sigma _{tot}^{2}=\sigma _{Poisson}^{2}+~\sigma _{dark}^{2}.\nonumber \end{align*}

Note that, under Poissonian noise statistics, the $\sigma _{Poisson}^{2}$ factor in equation (6) is simply the number of photons detected. The noise model described in equation (6) was added to the simulated images acquired at opposing edges at the 50% positions of the IC. Note that as the number of photons emitted by the x-ray source was kept constant for all system lengths (i.e. constant exposure kVp and mAs) the inverse square law was used to adjust the number of photons detected, using the 2 m standard EI XPCI setup as a reference, for the investigated system lengths. A normal distribution was assumed for DNS ($\sigma _{dark}^{2})$). The DNS was added to the noise model by attempting to find a single pair of values for DNS ($\sigma _{dark}^{2})$) and photon Poisson noise ($\sigma _{Poisson}^{2}$) that minimises the following chi-square function:

7 \begin{align*} \newcommand{\e}{{\rm e}} \displaystyle \chi _{(N_{ph}^{\left(2m \right)},dark)}^{2}=\sum\limits_{i=1}^{n}{\frac{({{e}_{i}}-{{s}_{i}}(N_{ph}^{\left(2m \right)},dark))}{{{s}_{i}}(N_{ph}^{\left(2m \right)},dark)}}~\nonumber \end{align*}

where ${{e}_{i}}$ is the set of experimental noise values, ${{s}_{i}}$ is the set of simulated ones which depend on the number of photons at 2 m ($N_{ph}^{\left(2m \right)})~$ and the DNS component (dark) used in equation (6) and *n*  =  5 is the number of investigated system lengths.

Four filaments of different composition and diameters were imaged: a nylon wire (‘Maxima’ brand) with a diameter of 295 *µ*m (*δ*  =  4.9  ×  10^−7^ and *β*  =  1.97  ×  10^−10^), polyetheretherketone (PEEK) with a diameter of 140 *µ*m (*δ*  =  5.3  ×  10^−7^ and *β*  =  2.2  ×  10^−10^ at 23 kV), polyethelene terephthalate (PET) with diameter of 100 *µ*m (*δ*  =  5.6  ×  10^−7^ and *β*  =  2.6  ×  10^−10^) and sapphire with a diameter of 240 *µ*m (*δ*  =  1.54  ×  10^−6^ and *β*  =  2.6  ×  10^−9^). The quoted *δ* and *β* values are for the estimated mean energy of the source operated at 40 kVp (23 kV) (Schoonjans *et al* 2011).

### 2.2. Computed-tomography acquisitions

Following initial analysis of the 2D images, CT acquisitions were performed at two system lengths; 0.85 m and 2 m. The 0.85 m system length was the shortest achievable due to the footprint of the motors we currently use; more compact motors would allow for even shorter system lengths. The source used for the CT acquisitions was a Rigaku 007-HF Micro Max (Rigaku Corporation, Japan) with a rotating molybdenum target and an effective focal spot size of approximately 70 *µ*m. Since we are ultimately targeting intraoperative breast specimen imaging, the choice of a molybdenum anode target was due to its optimum spectrum for the imaging of breast tissue. All CT acquisitions were made with 40 kVp and 20 mA source parameters. A continuous acquisition was performed over a total scan angle of 360° with 2500 projections and an exposure time of 1.44 s per projection. The same detector and masks as those used for the 2D acquisitions were employed. At each projection a single image acquisition was performed at the left slope of the illumination curve (see figure 1(b)). The acquired images were processed to obtain thickness map images which were then used to reconstruct cross sectional slices using a fan-beam reconstruction algorithm (Feldkamp *et al* 1984, Hagen *et al* 2014b, Diemoz *et al* 2016, 2017). The scanned samples were a water-filled plastic phantom of diameter 16 mm with a 5 mm diameter PMMA insert and an ethically approved 30 mm radius breast tissue specimen from the Barts tissue bank (St. Bartholomew’s Hospital, London UK). The specimen was fixed by paraformaldehyde immersion for 24 h.

### 2.3. Image analysis

The 2D images of the filaments were analysed to investigate the variation in noise and contrast to noise ratio (CNR) with total system length. Noise was measured as the standard deviation, *σ*, of pixel values in a background area adjacent to the filaments. This was performed over 5 different regions in order to obtain a standard deviation on the noise.

The CNR for refraction images (CNR*_ref_*) was calculated using

8 \begin{align*} \newcommand{\e}{{\rm e}} \displaystyle {\rm CN}{{{\rm R}}_{{ref}}}=~\frac{{{I}_{{\rm max}}}-{{I}_{{\rm min}}}}{\sigma }~\nonumber \end{align*}

where *I*_max_ and *I*_min_ are the maximum and minimum pixel values across the wire (averaged over the image FOV), respectively. CNR for absorption images (CNR_abs_) and thickness images (CNR_t_) were calculated using

9 \begin{align*} \newcommand{\e}{{\rm e}} \displaystyle {\rm CN}{{{\rm R}}_{{\rm abs/t}}}=~\frac{{{m}_{s}}-{{m}_{bkg}}}{\sigma }~\nonumber \end{align*}

where *m*_*s*_ and *m*_*bkg*_ are the mean pixel values in a small region of interest (ROI) in the central part of a filament and of the background (averaged over the FOV), respectively. CT images of the phantom were analysed for CNR_abs_ and CNR_t_ and the breast tissue specimen was only assessed qualitatively. It should be noted that the contrast obtained in refraction and absorption images are inherently two different quantities due to the difference in the obtained signal. A fairer comparison would be between CNR_abs_, CNR_t_ and the CNR obtained from phase shift (Φ) images (integrated refraction images). However, CNR_ref_ is discussed in this work to demonstrate the contribution of DNS in low dose acquisitions. Ultimately, we are interested in the comparison between CNR_t_ and CNR_abs_ because the speed requirements of intraoperative imaging are likely to prevent us from acquiring images on the two sides of the IC. Furthermore, other image quality metrics such as mean squared error (MSE) or structural similarity index (SSIM) which aim to approximate perceived visual quality would also be useful for comparison with theoretical profiles and will be considered for future work (Tan *et al* 2013).

## 3. Results and discussions

### 3.1. 2D: noise

**Figure 2.**
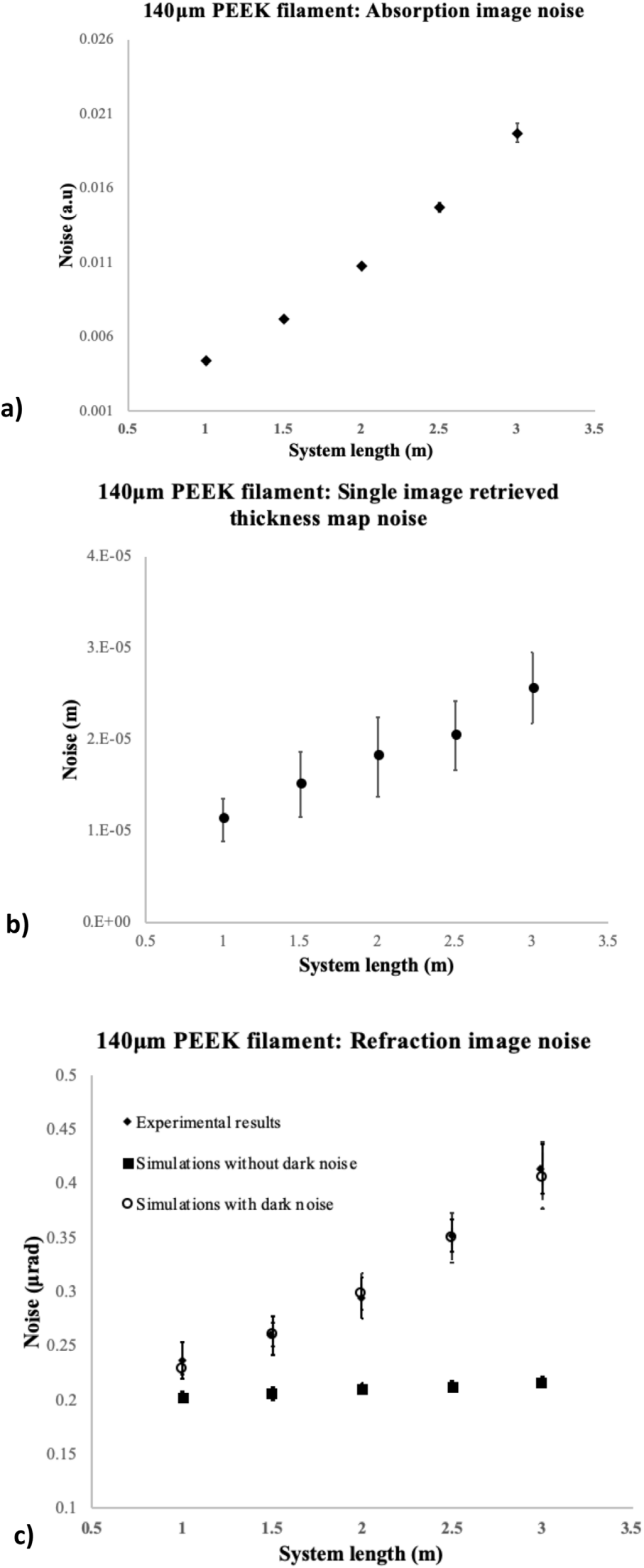
Noise measurements (taken as the standard deviation, *σ*, of pixel values in an area adjacent to the 140 *µ*m PEEK filament) for: (a) refraction image; experimental values are compared to those measured in simulations with and without accounting for the contribution of dark noise. (b) Thickness map images and (c) absorption images. Error bars indicate the standard deviation on the noise measurements obtained by taking measurements over 5 regions in the background area adjacent to the filament.

The effect of overall system size variation on the noise present in refraction, absorption and thickness map images of the 140 *µ*m PEEK filament is presented in figure 2. Similar trends were observed for all other filaments. Results for noise retrieved from acquired refraction images (figure 2(a)) are compared to simulated values. It can be seen that initial simulations, not accounting for the presence of DNS in integrating detectors, did not match the experimental results. In fact, initial simulations predicted a constant noise with increasing system length, in line with the prediction of equation (5). However, when a constant DNS component is added to the simulation’s noise model, this matched the experimental data well. Hence, it can be seen that, for constant exposure parameters, the relative contribution of the DNS to image noise increases with increasing system length. This can be mitigated by increasing the exposure time of a single frame. Figures 2(b) and (c) indicate the noise behaviour in thickness map and absorption images, respectively. The increase in noise is due to a combination of the decrease in number of photons incident on the detector following the inverse square law and DNS constribution. When comparing the noise results for the 1 m and 2 m system lengths, there was a 24%, 140% and 60% increase in image noise for the refraction, absorption and thickness map images, respectively.

### 3.2. 2D: contrast and CNR

**Figure 3.**
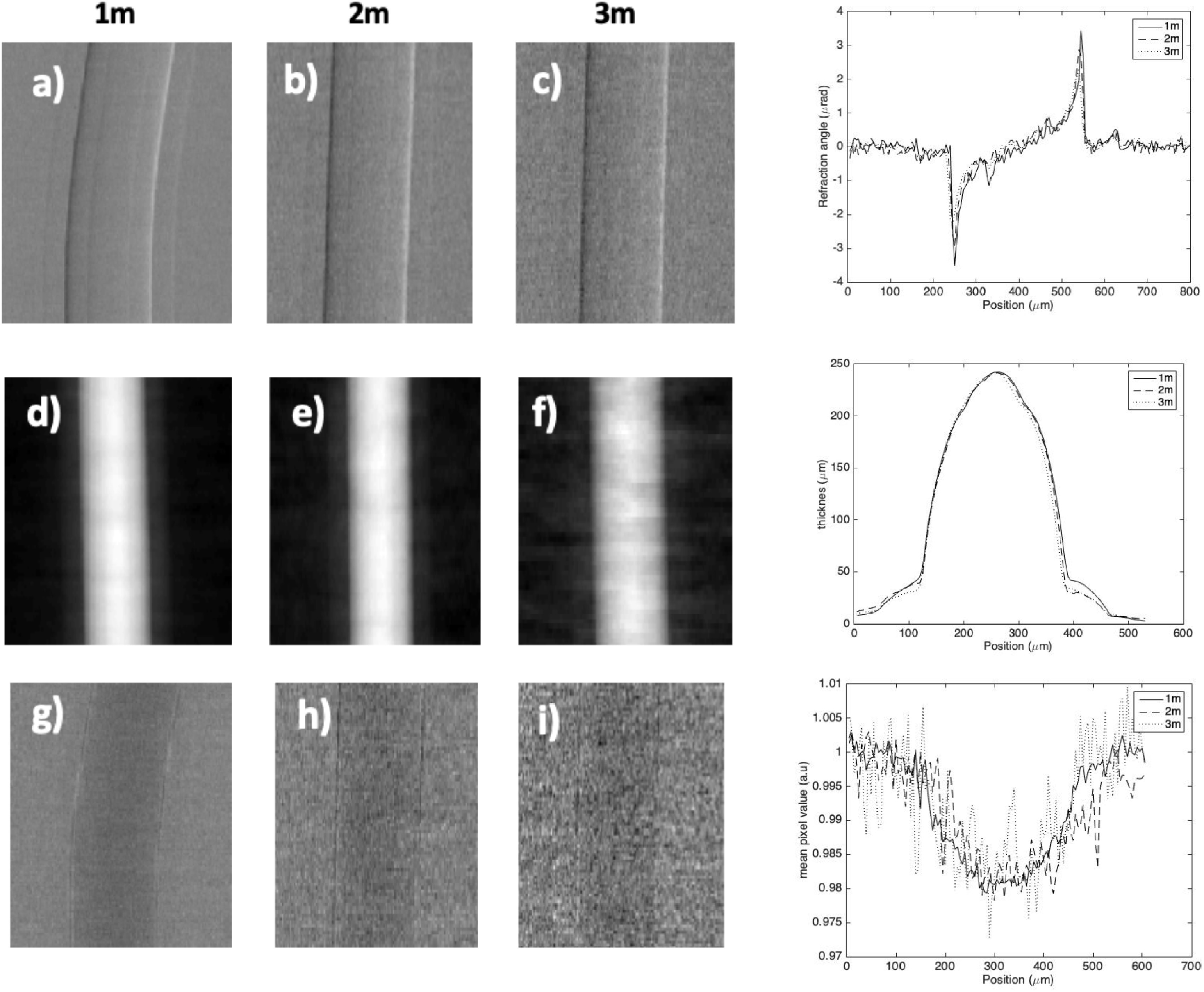
Refraction (a)–(c), thickness map (d)–(f) and absorption (g)–(i) images acquired at 1 m ((a), (d) and (g)), 2 m ((b), (e) and (h)) and 3 m ((c), (f) and (i)) system lengths and constant exposure parameters for a 250 *µ*m diameter sapphire filament. Plots on the right indicate corresponding mean intensity profiles across 10 rows along the centre of each image.

Reconstructed refraction, thickness map and absorption images with their corresponding intensity profiles for the 250 *µ*m sapphire filament wire acquired at 1 m, 2 m and 3 m system lengths can be seen in figure 3. For refraction images (figure 3(a)) a small decrease in contrast (*I*_max_–*I*_min_), with increasing system length, can be seen from both the images and the intensity profiles. As all parameters were kept constant this is believed to be due to a non perfectly parallel line-up between the source and detector, when increasing the system length, leading to a possible increase in the projected focal spot caused by the resulting difference in take off angle. An increase in projected source size leads to a broadening of the IC and hence a decrease in the reliability with which the refraction signal is retrieved.

**Figure 4.**
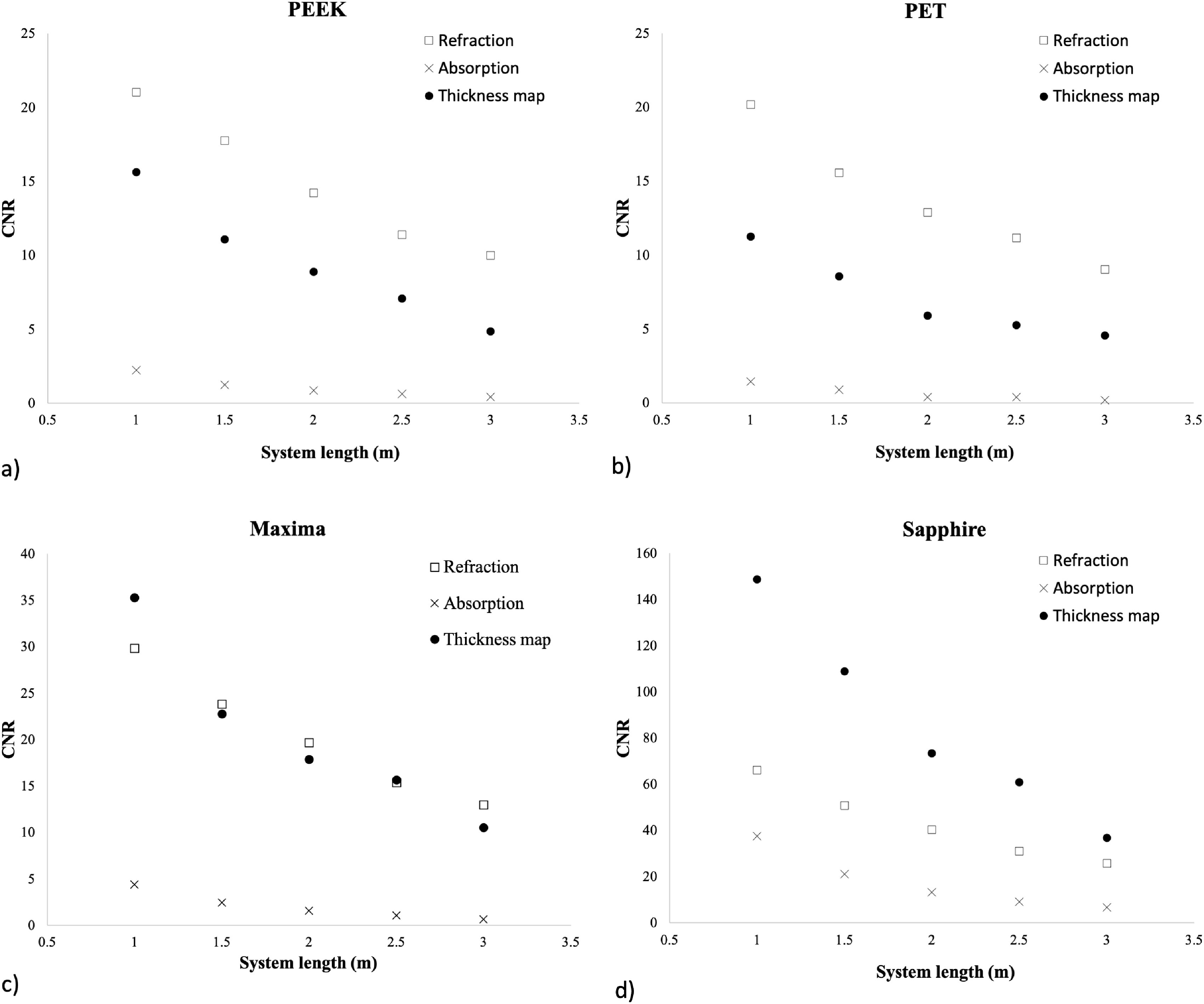
Contrast to noise ratio (CNR) measurements results with system length variation for refraction, absorption and thickness map reconstructed images of filament wires with varying composition and diameters: (a) 140 *μ*m polyetheretherketone (PEEK) (b) 140 *μ*m polyethelene terephthalate (PET), (c) 295 *μ*m Maxima and (d) 240 *μ*m Sapphire.

Results for CNR*_ref_*, CNR_abs_ and CNR_t_ measurements for the various filament compositions and diameters are presented in figure 4. Refraction and thickness map CNR results for all filaments were superior to those of the absorption images. The decrease in CNR with increasing system length is due to the increase in Poisson noise present in the images. CNR_t_ results for the sapphire filament were better than those of CNR_abs_ and CNR*_ref_*. This is believed to be due to a combination of the relatively thicker diameter of this filament and a lower *δ*/*β* ratio compared to the other filaments. CNR*_ref_* for the PEEK and PET filaments were consistently higher than both CNR_abs_ and CNR_t_ images. CNR*_ref_* and CNR_t_ measurements for the Maxima filament produced relatively similar results. Since the *δ*/*β* ratio of this filament is relatively similar to that of PEEK and PET, the improved CNR_t_ can be attributed to its thicker diameter. Results for the PEEK and PET filamentes are similar in all conditions due to their similar *δ* and *β* values. However, slightly better results can be seen for the PEEK CNR_t_ and CNR_abs_ values due to its thicker diameter. CNR improves on average by a factor 1.9 when comparing the 1m and 2m systems for all filaments.

### 3.3. CT CNR

**Figure 5.**
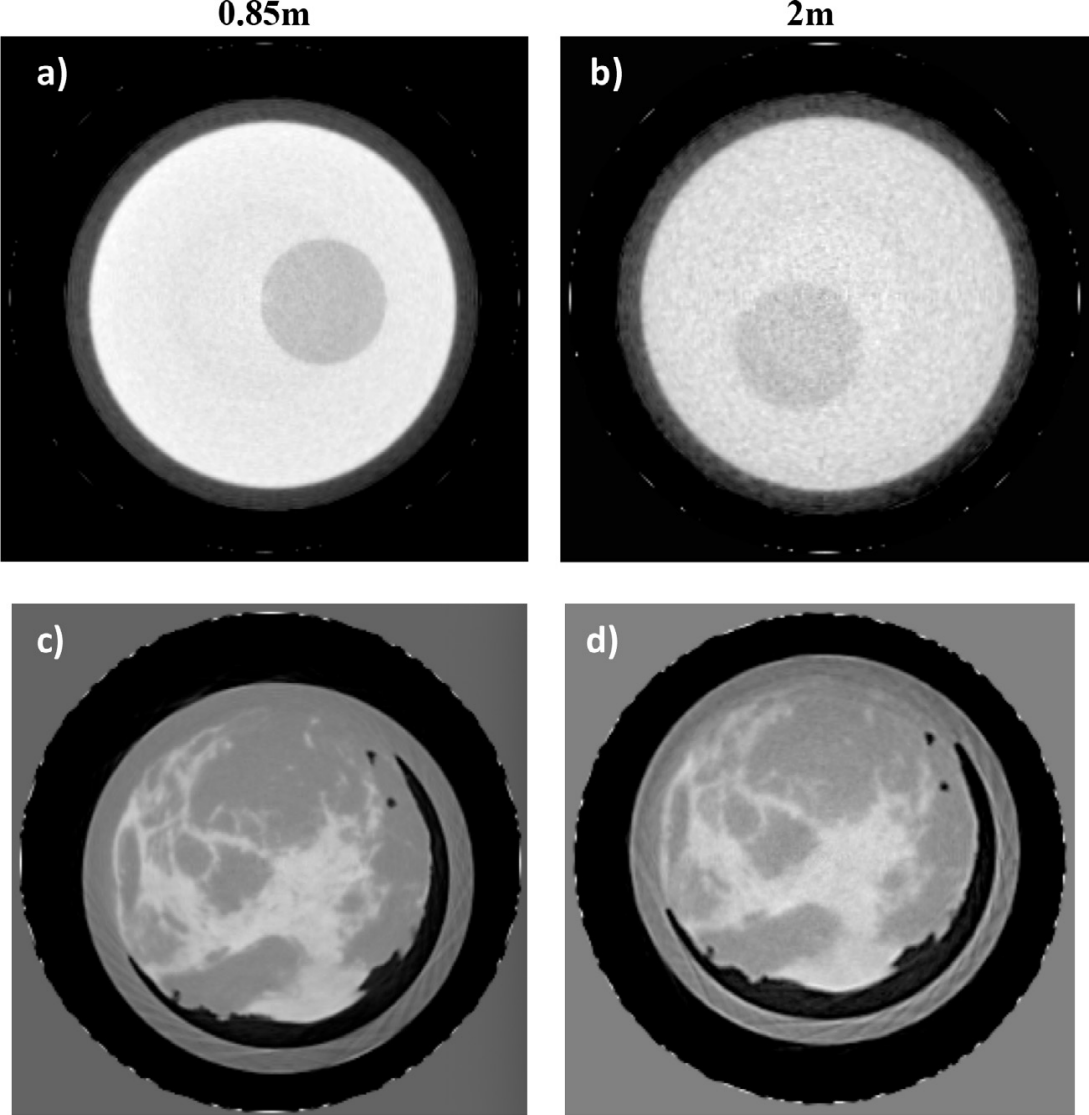
Computed Tomography (CT) transverse slices reconstructed from single shot images acquired at 0.85 m (left column) and 2 m (right column) total system lengths with constant exposure factors and magnification for a PMMA phantom ((a) and (b)) and an ethically approved, formalin fixed breast tissue specimen 30 mm in radius ((c) and (d)).

Transverse slices of the PMMA phantom and the breast tissue specimen obtained with the 0.85 m and 2 m CT setups can be seen in figure 5. CNR measurements for the PMMA phantom resulted in 8.8 and 2.6 for the 0.85 m and 2 m systems respectively. Thus, a more than two fold increase in CNR, is observed in line with the results obtained for the 2D acquisitions. It can be seen that this is also the case for the breast tissue specimen.

It should be noted that the CT acquisition duration of 1 h was due to limitations in the detector’s frame rate allowing only exposures at half the source power to avoid saturation. Faster acquisitions than the ones performed in this work can be achieved by using a detector with twice the frame rate, increasing the source output, shortening the system even more (by using motors with a smaller footprint) and decreasing the number of angular projections.

## 4. Conclusions

We have presented image noise results for three different image reconstructions obtained with the XPCI EI method implemented at multiple source to detector distances. We show that, when integrating detectors are used, the contribution of DNS to the noise present in images needs to be taken into account, especially for images acquired at relatively low photon statistics.

We show that refraction and thickness map images lead to improved CNR results when compared to absorption images both for relatively high and low attenuating materials. Results seem to suggest that for some materials, i.e. those with a low *δ*/*β* ratio (i.e. the ones for which a high absorption signal can be obtained) or thicker materials, thickness map images produce superior CNR results when compared to images containing only refraction signal. However, for a fairer comparison total phase shift images would have to be reconstructed instead of refraction ones. In principle, if only Poisson noise were present, i.e. when using a ‘perfect’ photon counter, then CNR_ref_ would be independent of source to detector distance for a given exposure time. However, in practice, when an integrating detector is used, the increasing relative weight of DNS makes shorter distances more advantageous, as the increased statistics for a constant exposure time makes it easier for the real counts to outweigh the dark noise ones. CNR_abs_ clearly improves with shorter distances because of the higher photon statistics. Due to time restrictions, the ‘single shot’ approach, which combines the refraction and absorption contrasts, is bound to be used for image acquisition in an intraoperative scanner. Hence the preference for a shorter system length is two fold, with one advantage coming from the higher contribution from CNR_abs_ and the other from the lower relative weight of DNS. Ultimately our experimental results confirm that in intraoperative specimen imaging shorter systems are not only feasible but preferable. The relative increase in radiation dose would not be an issue for a number of applications, among which ex vivo imaging of biological soft tissue as targeted here. Further work would be required to investigate the effects of using ‘skipped’ versus ‘non-skipped’ masks and reduction of scan times with the implementation of a detector with improved frame rate performance, higher source output and reduced angular projections. Furthermore, although the source parameters and detector intregration time implemented in this work were kept constant it is evident that faster acquisitions can be performed with short system setups if one would want to simply match, instead of exceed, image quality obtained with longer setups. Once a prototype system has been built an assessment of the system’s sensitivity and specificity will be performed with clinical specimens.
